# New Delhi Metallo-β-Lactamase 5–Producing *Klebsiella pneumoniae* Sequence Type 258, Southwest China, 2017

**DOI:** 10.3201/eid2506.181939

**Published:** 2019-06

**Authors:** Xin Zhang, Weimin Wan, Hua Yu, Min Wang, Haifang Zhang, Jingnan Lv, Yi-Wei Tang, Barry N. Kreiswirth, Hong Du, Liang Chen

**Affiliations:** Sichuan Academy of Medical Science and Sichuan Provincial People’s Hospital, Sichuan (X. Zhang, H. Yu);; Memorial Sloan Kettering Cancer Center, New York, New York, USA (X. Zhang, Y.-W. Tang);; The Second Affiliated Hospital of Soochow University, Suzhou, China (W. Wang, M. Wang, H. Zhang, J. Lv, H. Du);; Rutgers University, Newark, New Jersey, USA (B.N. Kreiswirth, L. Chen)

**Keywords:** New Delhi metallo-β-lactamase 5, NDM-5 gene, Klebsiella pneumoniae, bacteria, antimicrobial resistance, sequence type 258, ST258, carbapenem-resistant K. pneumoniae, plasmid, horizontal transfer, molecular evolution, China

## Abstract

We isolated a New Delhi metallo-β-lactamase 5 (NDM-5)–producing *Klebsiella pneumoniae* sequence type (ST) 258 strain in southwest China during 2017. The *bla*_NDM-5_ gene was acquired by horizontal plasmid transfer from NDM-5–producing *Escherichia coli*. We identified genomic characteristics in ST258 strains that differed from those of global *K. pneumoniae* carbapenemase–producing strains.

Carbapenem-resistant *Klebsiella pneumoniae* has emerged as one of the major multidrug-resistant bacterial pathogens worldwide. The international spread of this pathogen has been linked to a few high-risk clone group (CG) strains, including CG258, CG14/15, CG17/20, CG43, CG147, and CG307 (*1*), of which CG258 is the most widely spread. Within CG258, sequence type (ST) 258 predominates in North America and Europe, but ST11 is the primary carbapenem-resistant *K. pneumoniae* clone in eastern Asia, especially in China (*2*–*4*).

In recent years, *K. pneumoniae* ST11 strains have been increasingly identified in North America. In contrast, ST258 strains are still rarely reported in China (*5*). Unlike other high-risk clones, such as ST11, that harbor different types of carbapenemases, carbapenem-resistant *K. pneumoniae* ST258 are almost exclusively associated with *K. pneumoniae* carbapenemase (KPC) (*6*). We report identification of a New Delhi metallo-β-lactamase 5 (NDM-5)–producing *K. pneumoniae* ST258 strain from Chengdu in southwest China.

## The Study

In June 2017, a *K. pneumoniae* strain (Kp2588) that has an extended-spectrum β-lactamase phenotype was isolated from a blood culture of a male patient in a large tertiary care hospital in Chengdu in southwest China. Two days later, a carbapenem-resistant *Escherichia coli* strain (Ec2551) was isolated from a urine culture, and 1 week later, a carbapenem-resistant *K. pneumoniae* strain (Kp2573) was isolated from another urine culture, both from the same patient (Appendix).

PCR testing and Sanger sequencing showed that Ec2551 and Kp2573 both harbored the New Delhi metallo-β-lactamase gene *bla*_NDM-5_. Multilocus sequence typing further showed that Ec2551 is an ST48 strain, and Kp2588 and Kp2573 belong to the high-risk clone ST258.

Susceptibility testing showed that the initial Kp2588 blood isolate was resistant to most cephalosporins and aztreonam but susceptible to cefepime, carbapenems, amoxicillin/clavulanic acid, piperacillin/tazobactam, amikacin, and tigecycline (Table). In contrast, the urinary isolates Ec2551 and Kp2573 were resistant to all β-lactams and β-lactam/β-lactamase inhibitors, including the 4 carbapenems tested in this study (Table).

**Table:** Antimicrobial drug susceptibility of 3 bacterial isolates producing New Delhi metallo-β-lactamase 5, southwest China, 2017*

Characteristic	Species, strain, and MIC, mg/L		
	*Klebsiella pneumoniae*, Kp2588	*Escherichia coli*, Ec2551	*K. pneumoniae*, Kp2573
Source	Blood	Urine	Urine
Sequence type	258	48	258
Drug			
Meropenem	≤0.25	**≥16**	**≥16**
Imipenem	≤0.25	**≥16**	**≥16**
Doripenem	≤0.12	**≥8**	**≥8**
Ertapenem	≤0.5	**≥8**	**≥8**
Ampicillin	**≥32**	**≥32**	**≥32**
Ticarcillin	**≥128**	**≥128**	**≥128**
Cephalothin	**≥64**	**≥64**	**≥64**
Cefazolin	**≥64**	**≥64**	**≥64**
Cefuroxime	**≥64**	**≥64**	**≥64**
Ceftazidime	16	**≥64**	**≥64**
Cefotaxime	**≥64**	**≥64**	**≥64**
Ceftriaxone	**≥64**	**≥64**	**≥64**
Cefpodoxime	**≥8**	**≥8**	**≥8**
Ceftizoxime	≤1	**≥64**	**≥64**
Cefepime	2	**≥64**	**≥64**
Cefotetan	≤4	**≥64**	**≥64**
Aztreonam	**≥64**	**≥64**	**≥64**
Piperacillin	**≥128**	**≥128**	**≥128**
Ampicillin/sulbactam	**≥32/16**	**≥32/16**	**≥32/16**
Amoxicillin/clavulanic acid	16/8	**≥32/16**	**≥32/16**
Piperacillin/tazobactam	64/4	**≥128/4**	**≥128/4**
Nitrofurantoin	**256**	≤16	**256**
Gentamicin	**≥16**	**≥16**	**≥16**
Tobramycin	**≥16**	**≥16**	**≥16**
Amikacin	4	≤2	4
Ciprofloxacin	**≥4**	**≥4**	**≥4**
Levofloxacin	**≥8**	**≥8**	**≥8**
Moxifloxacin	**≥8**	**≥8**	**≥8**
Trimethoprim/sulfamethoxazole	**≥16/304**	**≥16/304**	**≥16/304**
Nalidixic acid	**≥32**	**≥32**	**≥32**
Tetracycline	**≥16**	**≥16**	**≥16**
Tigecycline	2	≤0.5	2

*Drug-resistant MICs are indicated in bold. Antimicrobial susceptibility testing was conducted by using the VITEK 2 system (bioMérieux, https://www.biomerieux.com).

**Figure 1:**
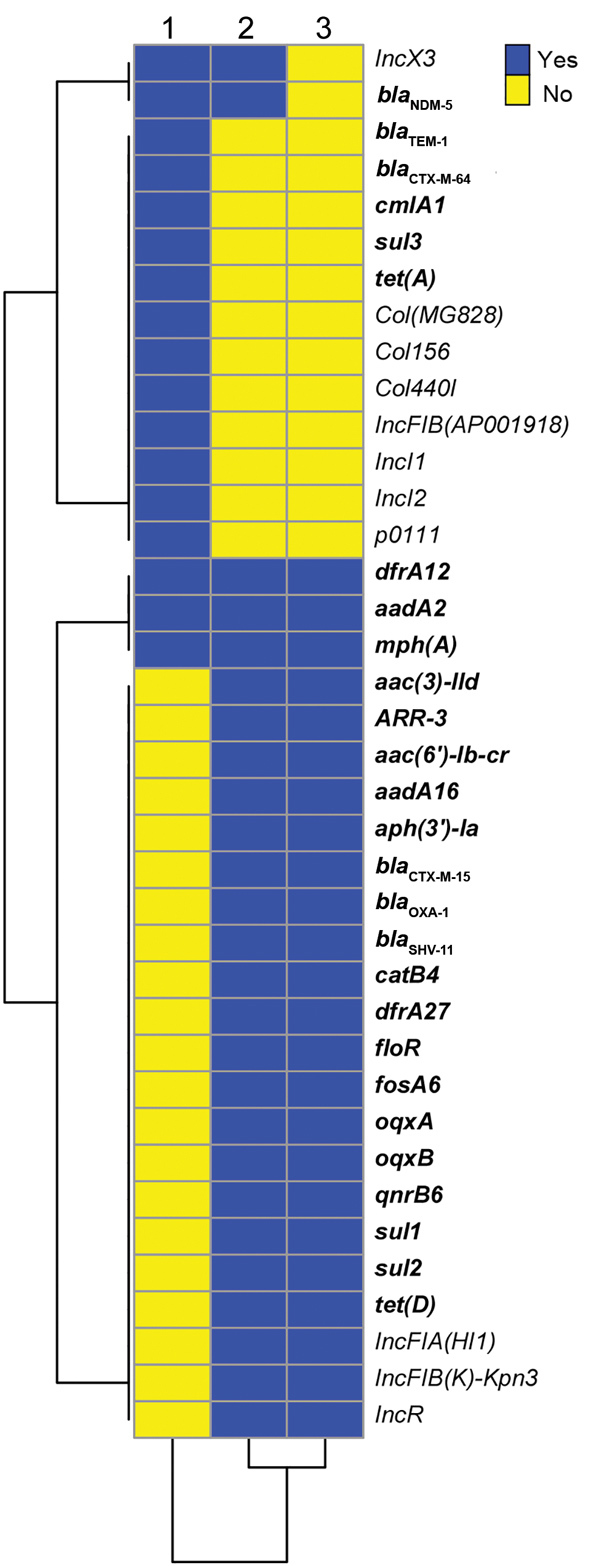
Comparison of resistance genes and plasmid replicons for 3 bacterial isolates producing New Delhi metallo-β-lactamase 5, southwest China, 2017. 1, *Escherichia coli* 2551; 2, *Klebsiella pneumoniae* 2573; 3, *K. pneumoniae* 2588. Resistance genes are shown in bold.

**Figure 2:**
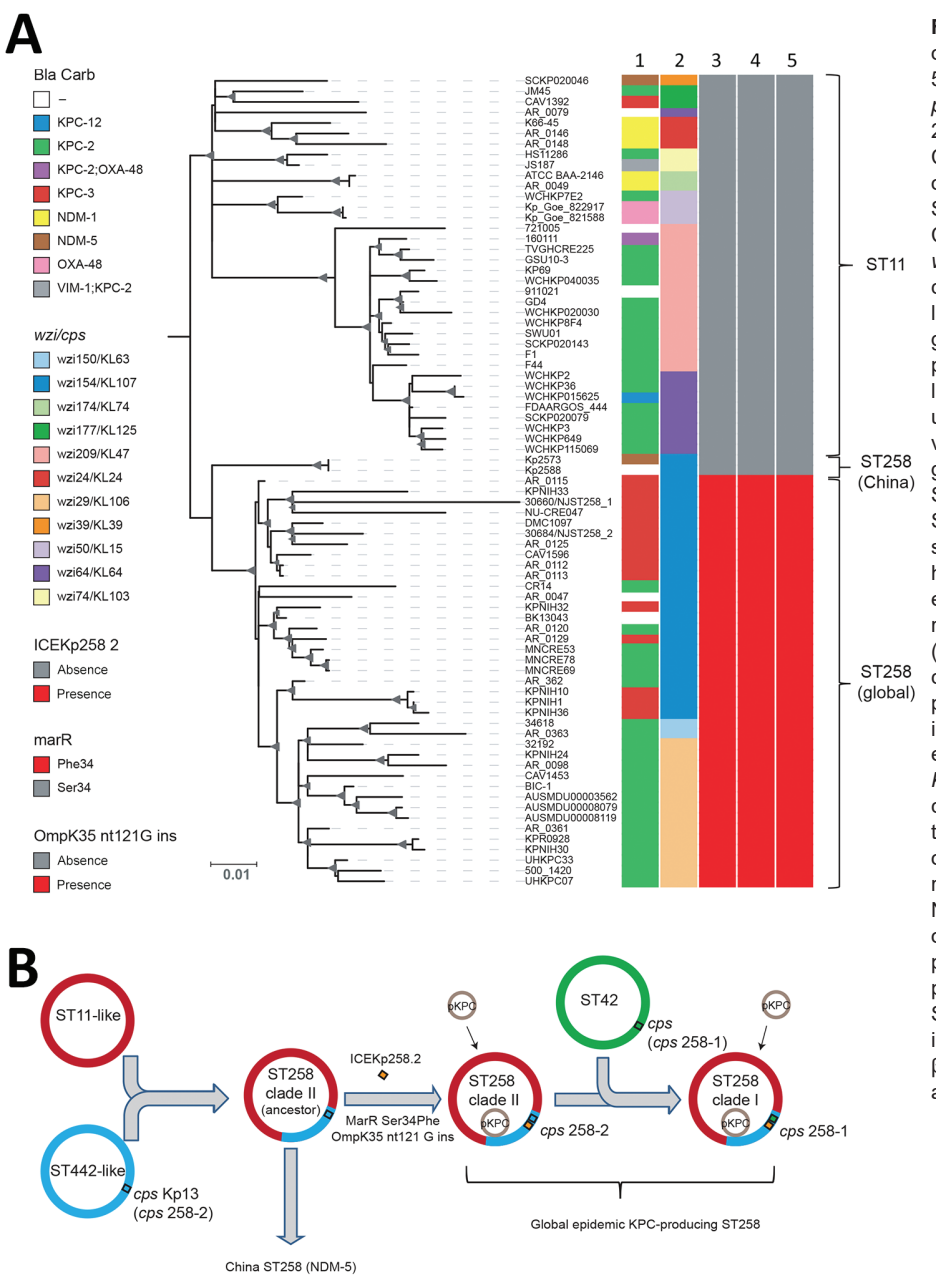
Phylogenetic analysis of KPC-producing and NDM-5–producing CG258 *Klebsiella pneumoniae* strains from China, 2017, and reference strains. A) Core SNP phylogenetic analysis of 76 global CG258 (ST258 and ST11) and 2 ST258 strains from China. Lane 1, Bla_carb; lane 2, *wzi* (cps); lane 3, integrative and conjugative element Kp258.2; lane 4, *marR*; lane 5, *omp*K35 gene (guanine insertion at nt position 121). The maximum-likelihood tree was rooted by using ST11 strains. Bootstrap values >90% are indicated as gray triangles at branch points. Sizes are proportion to values. Scale bar indicates nucleotide substitutions per site. B) Updated hypothesis of the molecular evolution of carbapenem-resistant *K. pneumoniae* ST258 (*9*). Bla, β-lactamase; Carb, carbapenemase; *cps*, capsular polysaccharide gene; ICE, integrative and conjugative element; ins, insertion; KPC, *Klebsiella pneumoniae* carbapenemase; MarR, transcriptional regulator protein of the multiple antimicrobial drug resistance repressor family; NDM, New Delhi metallo-β-lactamase; ompK35, outer membrane protein K35; OXA, oxacillinase; pKPC, plasmid carrying KPC; ST, sequence type; VIM-1, Verona integron–encoded metallo- β-lactamase 1; *wzi*, surface assembly of capsule gene.

We conducted next-generation sequencing (NGS; Illumina Hiseq, https://www.illumina.com) for all 3 isolates and deposited data in the National Center for Biotechnology (NCBI) Bioproject PRJNA354234 (https://www.ncbi.nlm.nih.gov/bioproject) GenBank database (accession nos. RXHE00000000, RXHG00000000 and RXHF00000000, respectively). Analysis of resistance genes and plasmid sequences showed that the 2 ST258 *K. pneumoniae* isolates carried similar resistance and plasmid replicon genes, except that Kp2573 harbored *bla*_NDM-5_ and an IncX3 plasmid replicon (Figure 1). *E. coli* strain Ec2551 had the same *bla*_NDM-5_ and IncX3 replicon genes as Kp2573, but also had a diverse array of other resistance and plasmid replicon genes in comparison with the 2 *K. pneumoniae* isolates (Figure 2).

We then transferred *bla*_NDM-5_–harboring plasmids from Ec2551 and Kp2573 to recipient strain *E. coli* J53^AZR^ by conjugation, followed by plasmid NGS as described. Plasmids sequence analysis showed that Ec2551 and Kp2573 carry an identical *bla*_NDM-5_–harboring IncX3 plasmid. The plasmid is 46,161 bp and is identical to several *bla*_NDM-5_–harboring plasmids identified in China and other countries (e.g., GenBank accession nos. CP032424, MF679143, MG591703, CP028705, and CP024820). Core single-nucleotide polymorphism (SNP) analysis showed that Kp2588 differed from Kp2573 by 1 SNP. These results strongly suggest that carbapenem-resistant Kp2573 might have evolved from the carbapenem-susceptible blood isolate Kp2588 by acquisition of a *bla*_NDM-5_–harboring IncX3 plasmid from the same patient.

To date, carbapenemases other than KPC have rarely been described in clinical *K. pneumoniae* ST258 strains (*7*,*8*). In this study, we had the opportunity to investigate the phylogenetic relationship between NDM-5–producing ST258 (designated as the China ST258 clone) and other global KPC-producing ST258 strains (Figure 2, panel A). Phylogenetic analysis of core SNPs from the 2 China ST258 genomes and 76 completely closed ST258 (n = 39) and ST11 (n = 36) genomes from NCBI showed that Kp2573/Kp2588 belongs to a separate clade distinct from global ST258 and ST11 strains (Figure 2, panel A). Kp2573/Kp2588 differs from global ST258 genomes by an average of 88 core SNPs (range 54–135) and from ST11 strains by an average of 109 core SNPs (range 79–132).

Results indicated that Kp2573/Kp2588 and the global ST258 strains both evolved from a common ancestor. A different core SNP phylogenetic analysis of Kp2573/Kp2588 with 824 ST258 genomes (including both draft assemblies and completely closed genomes) from NCBI showed similar results, suggesting that Kp2573/Kp2588 has unique genetic characteristics in comparison with other global ST258 strains.

Recent phylogenetic analysis showed that global ST258 strains have unique genetic characteristics that might contribute to their epidemic spread. For example, global ST258 strains carry a unique integrative and conjugative element (ICE) known as ICEKp258.2 (*3*). This chromosomal element is conserved among nearly all ST258 strains, harboring a type IV pilus gene cluster and a type III restriction-modification system, which might contribute to plasmid acquisition or plasmid–host specificity (*9*). However, ICEKp258.2 is absent from genomes of Kp2573/Kp2588 (Figure 2, panel A). In addition, global ST258 strains have a common Ser34Phe amino acid substitution in the homodimerization region of MarR, a transcriptional regulator protein of the multiple antimicrobial drug resistance repressor family (*10*). It is hypothesized that this substitution might affect the overall metabolic activity in ST258 (*10*). Kp2573/Kp2588 has the ancestral MarR Ser34 genotype, similar to ST11 strains, but all global ST258 strains have the Phe34 genotype. Furthermore, all global ST258 strains contain a frame-shift mutation that results in a premature stop codon at amino acid position 89 in outer membrane protein OmpK35 because of a guanine insertion at nt position 121. In contrast, Kp2573/Kp2588 do not have the nt121G insertion in the *ompK35* gene but have an IS*1* insertion at nt position 28. These genetic changes further suggest that the 2 China ST258 strains have distinct evolutionary paths compared with those of global ST258 strains.

These observations have also updated our previous hypothesis regarding the molecular evolution of ST258 (Figure 2, panel B) (*9*). Our initial study suggested that the prototypical ST258 strain arose as a consequence of recombination of large segments of the ST11 and ST442 genomes, such that it has the same *wzi154*/KL107 (previously named *cps-2*/clade II) capsular type as the parental ST442 strain (*9*). Additional genotypic changes, including acquisition of ICEKp258.2, the MarR Ser34Phe substitution, and the *ompK35* nt121G insertion, as well as the acquisition of KPC plasmids in ST258 clade II strains, define the global KPC-producing carbapenem-resistant *K. pneumoniae* ST258 clone.

A second molecular event, generated by replacement of the ST258 clade II *cps* region with the corresponding region from ST42, subsequently created the equally prevalent ST258 clade I clone (*9*). Both clades I and II ST258 strains have shown the ability to host diverse KPC plasmids, which might in part contribute to the spread of global ST258 strains. In contrast, the China ST258 strains appear to have evolved separately, recently acquiring the *bla*_NDM5_–harboring plasmid (Figure 2, panel B), highlighting the continuing evolution of *K. pneumoniae* ST258 strains.

## Conclusions

We report the genotype of an NDM-5–producing *K. pneumoniae* ST258 strain isolated in southwest China. The *bla*_NDM-5_ gene was likely acquired by a carbapenem-susceptible ST258 strain from an NDM-5–producing *E. coli* strain in vivo in the same patient as a result of horizontal transfer of a *bla*_NDM-5_–harboring IncX3 plasmid. Genomic comparison of the ST258 strain from China with other carbapenem-resistant *Klebsiella pneumoniae* ST258 strains indicated that they both evolved from a common ancestral strain but with distinct genetic characteristics suggestive of separate evolutionary histories. Further genomic comparisons between ST258 strains from China and their global ST258 counterparts will help elucidate the molecular mechanisms underscoring the spread of this high-risk clone.

Appendix Additional information on New Delhi metallo-β-lactamase 5–producing *Klebsiella pneumoniae* sequence type 258, southwest China, 2017.
