# Supplementary material for: New Delhi Metallo-β-Lactamase 5–Producing Klebsiella pneumoniae Sequence Type 258, Southwest China, 2017
Source: Emerg Infect Dis. 2019 Jun;25(6):1209–13. doi: 10.3201/eid2506.181939 (PMC6537745; doi:10.3201/eid2506.181939)
Supplement: Appendix — Additional information on New Delhi metallo-β-lactamase 5–producing Klebsiella pneumoniae sequence type 258, southwest China, 2017. [file 18-1939-Techapp-s1.pdf]

Article DOI: https://doi.org/10.3201/eid2506.181939

New Delhi Metallo-β-Lactamase 5–Producing *Klebsiella pneumoniae* Sequence Type 258, Southwest China, 2017

Appendix

Case Description

In June 2017, a man in his middle 70s was given a diagnosis of bladder cancer. He underwent laparoscopic radical cystectomy and ileal neobladder reconstruction according to the da Vinci Surgical System (https://www.davincisurgery.com) in a large tertiary care hospital in Chengdu, southwest China. The patient had no history of travel during the past 10 years outside the province where he lived. However, he reported previous hospitalizations in numerous local healthcare centers.

One week after the surgery, the patient had diarrhea, nausea, and vomiting, and a subsequent stool sample positive for *Clostridioides difficile* toxin A/B, which was suggestive of a *C. difficile* infection. After he was given vancomycin and metronidazole for 9 days, the assay result was negative for *C. difficile* toxin A/B. However, bacteremia developed in the patient, and an extended-spectrum-β-lactamase phenotype *Klebsiella pneumoniae* (Kp2588) was isolated from a blood culture. The patient was then given piperacillin/tazobactam and moxifloxacin. Two days later, a carbapenem-resistant *Escherichia coli* (Ec2551) was recovered from a urine culture, although no major urinary tract symptoms were observed. One week later, the patient had symptoms of a severe urinary tract infection and a high fever (41°C). Subsequently, a carbapenemase-resistant *K. pneumoniae* strain (Kp2573) was recovered from a urine culture. His drug regimen was then changed to tigecycline and later with a combination of fosfomycin and amikacin.

Two weeks later, the carbapenemase-resistant *K. pneumoniae* appeared to have been cleared from the urinary tract. However, pneumonia developed, and extended-spectrum-β-

lactamase–positive *K. pneumoniae*, *Acinetobacter baumannii,* methicillin-resistant *Staphylococcus aureus*, and *Candida albicans* were isolated from multiple respiratory tract samples. He also had a high fever. One month later, the health of the patient condition continued to deteriorate. The patient then refused further treatment and requested to be discharged.

Whole-Genome Sequence Analysis

We sequenced 3 isolates by using the Hiseq platform (Illumina, https://www.illumina.com), which produced an average of 9.7 million (range 4.7 million–12.7 million) paired end reads of 150 bp in forward and reverse directions with a predicted minimum coverage of 200×. Illumina raw reads from whole-genome sequencing were trimmed by using Trimmomatic version 0.36 (*1*), followed by de novo assembly using SPAdes version 3.11.1 (*2*). We determined the multilocus sequence type in silico (https://github.com/tseemann/mlst) and identified acquired drug resistance genes and plasmid replicons by using Abricate (https://github.com/tseemann/abricate) and ARIBA (*3*) on the basis of the ResFinder (*4*) and PlasmidFinder (*5*) databases. For bla_{NDM-5}–harboring plasmid sequencing, we extracted plasmid DNA from *E. coli* J53 transconjugants and sequenced them by using the Illumina Hiseq system. We de novo assembled sequencing reads by using SPAdes version 3.11.1 (*2*). We closed gaps between contigs by using a PCR, followed by standard Sanger sequencing.

The genomes sequenced in this study (Kp2573 and Kp2588) were compared with genomes deposited in the National Center for Biotechnology Information assembly database (https://www.ncbi.nlm.nih.gov/assembly). All genomes were mapped to the *K. pneumoniae* reference genome NJST258_2 (GenBank accession no. CP006918) by using Snippy (https://github.com/tseemann/snippy). Prophages were predicted by using PHASTER (*6*), repeated region were examined by using MUMmer (*7*), and putative regions of recombination were predicted by using Gubbins (*8*), followed by filter-using vcftools (*9*). A recombination-free single-nucleotide polymorphism (SNP) phylogenetic tree was generated by using RAxML version 8.2.4 (https://cme.h-its.org/exelixis/web/software/raxml/) and a general time reversible model of nucleotide substitution with a Gamma model of rate heterogeneity and 4 rate categories (*10*). SNPs were annotated by using SnpEff 4.3 (*11*). Phylogenetic clades were identified by using hierarchical Bayesian analysis of population structure. The phylogenetic tree was annotated by using iTOL (*12*).

A different core SNPs phylogenetic analysis of Kp2573/Kp2588 and 824 ST258 genomes (including completely closed and draft assemblies) from the National Center for Biotechnology Information (https://www.ncbi.nih.gov) showed similar results because Kp2573/Kp2588 is distinct from global ST258 strains. However, Kp2573 and Kp2588 have the same ancestor, suggesting that Kp2573/Kp2588 carry some unique genetic characteristics in comparison with other global ST258 strains.

Nucleotide Sequence Accession Numbers

Draft genome assemblies and raw reads from our study were deposited in GenBank Bioproject No. PRJNA354234. The draft genome sequences of the 3 strains isolated in this study were deposited in the GenBank whole-genome shotgun database under accession nos, RXHE00000000, RXHG00000000, RXHF00000000, respectively. The raw reads were deposited in sequence read archive database under biosample accession nos. SAMN10583475, SAMN10583476, and SAMN10583477, respectively.

References

1. Bolger AM, Lohse M, Usadel B. Trimmomatic: a flexible trimmer for Illumina sequence data. Bioinformatics. 2014;30:2114–20. PubMed http://dx.doi.org/10.1093/bioinformatics/btu170

2. Bankevich A, Nurk S, Antipov D, Gurevich AA, Dvorkin M, Kulikov AS, et al. SPAdes: a new genome assembly algorithm and its applications to single-cell sequencing. J Comput Biol. 2012;19:455–77. PubMed http://dx.doi.org/10.1089/cmb.2012.0021

3. Hunt M, Mather AE, Sánchez-Busó L, Page AJ, Parkhill J, Keane JA, et al. ARIBA: rapid antimicrobial resistance genotyping directly from sequencing reads. Microb Genom. 2017;3:e000131. PubMed http://dx.doi.org/10.1099/mgen.0.000131

4. Zankari E, Hasman H, Cosentino S, Vestergaard M, Rasmussen S, Lund O, et al. Identification of acquired antimicrobial resistance genes. J Antimicrob Chemother. 2012;67:2640–4. PubMed http://dx.doi.org/10.1093/jac/dks261

5. Carattoli A, Zankari E, García-Fernández A, Voldby Larsen M, Lund O, Villa L, et al. In silico detection and typing of plasmids using PlasmidFinder and plasmid multilocus sequence typing. Antimicrob Agents Chemother. 2014;58:3895–903. PubMed http://dx.doi.org/10.1128/AAC.02412-14

6. Arndt D, Grant JR, Marcu A, Sajed T, Pon A, Liang Y, et al. PHASTER: a better, faster version of the PHAST phage search tool. Nucleic Acids Res. 2016;44:W16-21. PubMed http://dx.doi.org/10.1093/nar/gkw387

7. Kurtz S, Phillippy A, Delcher AL, Smoot M, Shumway M, Antonescu C, et al. Versatile and open software for comparing large genomes. Genome Biol. 2004;5:R12. PubMed http://dx.doi.org/10.1186/gb-2004-5-2-r12

8. Croucher NJ, Page AJ, Connor TR, Delaney AJ, Keane JA, Bentley SD, et al. Rapid phylogenetic analysis of large samples of recombinant bacterial whole genome sequences using Gubbins. Nucleic Acids Res. 2015;43:e15. PubMed http://dx.doi.org/10.1093/nar/gku1196

9. Danecek P, Auton A, Abecasis G, Albers CA, Banks E, DePristo MA, et al.; 1,000 Genomes Project Analysis Group. The variant call format and VCFtools. Bioinformatics. 2011;27:2156–8. PubMed http://dx.doi.org/10.1093/bioinformatics/btr330

10. Stamatakis A. RAxML version 8: a tool for phylogenetic analysis and post-analysis of large phylogenies. Bioinformatics. 2014;30:1312–3. PubMed http://dx.doi.org/10.1093/bioinformatics/btu033

11. Cingolani P, Platts A, Wang L, Coon M, Nguyen T, Wang L, et al. A program for annotating and predicting the effects of single nucleotide polymorphisms, SnpEff: SNPs in the genome of *Drosophila melanogaster* strain w1118; iso-2; iso-3. Fly (Austin). 2012;6:80–92. PubMed http://dx.doi.org/10.4161/fly.19695

12. Letunic I, Bork P. Interactive tree of life (iTOL) v3: an online tool for the display and annotation of phylogenetic and other trees. Nucleic Acids Res. 2016;44:W242-5. PubMed http://dx.doi.org/10.1093/nar/gkw290
